# Crystallographic Disorder and Strong Magnetic Anisotropy in Dy_3_Pt_2_Sb_4.48_

**DOI:** 10.1021/acs.inorgchem.3c01850

**Published:** 2024-02-14

**Authors:** Terry Paske, Yingdong Guan, Chaoguo Wang, Curtis Moore, Zhiqiang Mao, Xin Gui

**Affiliations:** † Department of Chemistry, University of Pittsburgh, Pittsburgh, Pennsylvania 15260, United States; ‡ Department of Physics, Pennsylvania State University, University Park, Pennsylvania 16801, United States; § Department of Chemistry and Biochemistry, The Ohio State University, Columbus, Ohio 43210, United States

## Abstract

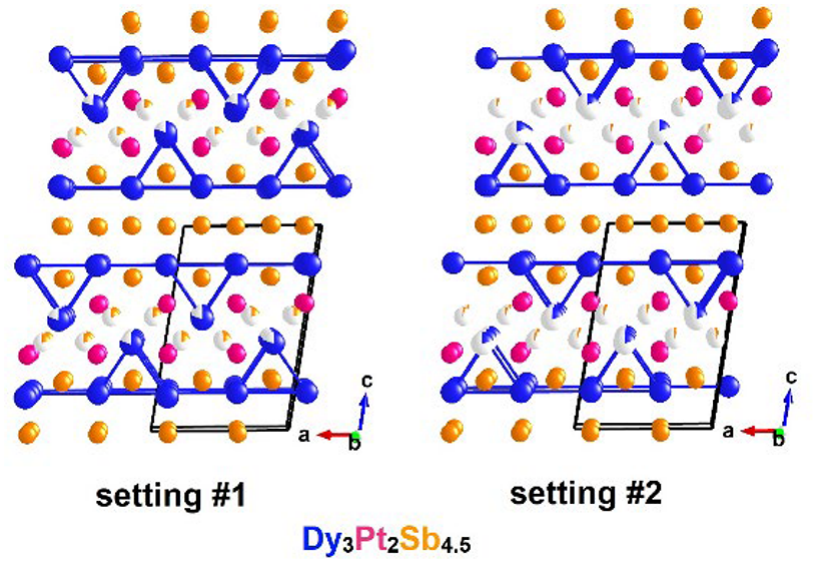

We report the crystal growth and characterization of a rare-earth-containing material, Dy_3.00(1)_Pt_2_Sb_4.48(2)_. This compound possesses a similar structure to the previously reported Y_3_Pt_4_Ge_6_, but it lacks two layers of Pt atoms. Crystallographic disorder was found in Dy_3.00(1)_Pt_2_Sb_4.48(2)_. Additionally, the Dy–Dy framework was found to have both square net and triangular lattices. Dy_3.00(1)_Pt_2_Sb_4.48(2)8_ was determined to be antiferromagnetically ordered around ∼15 K while a competing antiferromagnetic sublattice also exists at lower temperature. Strong magnetic anisotropy was observed, and several metamagnetic transitions were seen in the hysteresis loops. Furthermore, the Curie–Weiss fitting revealed an unusually small effective moment of Dy, which is far below the expected value of Dy^3+^ (10.65 μ_B_). This material might provide a new platform to study the relationship between crystallographic disorder and magnetism.

## Abstract 2

Single crystals of a new material, Dy_3_Pt_2_Sb_4.48_, were synthesized and characterized. Crystal defects and magnetic anisotropy were found in the new compound. It could be treated as a combination of two settings of defected structures where Dy atoms formed distorted square net and triangular lattices. Low-temperature antiferromagnetic transition was observed with an unconventionally low effective moment of Dy being found.

## Introduction

Rare-earth-based materials are of great importance for the investigation of frustrated magnets since they possess highly localized moments and have the ability to crystallize into various structural types. It is well known that magnetic frustration commonly results from the competing antiferromagnetic (AFM) interactions between nearest neighbors or even next-nearest neighbors.^1,2^ To allow such competition, geometric frustration of the sublattice of magnetic atoms is one of the most important routes to realizing frustrated magnetism. The most widely investigated frustrated structural motifs include the triangular lattice (YbMgGaO_4_),^3−6^ Kagome lattice (ZnCu_3_(OH)_6_Cl_2_),^3,7−9^ and pyrochlore/breathing pyrochlore lattice (Ce_2_Sn_2_O_7_/Ba_3_Yb_2_Zn_5_O_11_).^10−14^ These structural motifs are all composed of triangles. Ideally, equilateral triangles should exist in such systems, and their frustration can be “isotropic”. However, square lattices were also found to be great motifs for magnetic frustration since they can be seen as two edge-sharing isosceles right triangles.^15−17^ Thus, when the competing exchange coupling between the nearest neighbors (*J*_1_) and the next-nearest neighbors (*J*_2_) falls into an appropriate region, magnetic frustration can be expected.^18^

Y_3_Pt_4_Ge_6_ was reported to crystallize in the *P*2_1_/*m* space group.^19^ In recent years, the crystal structure and magnetic properties of polycrystalline Ln_3_Pt_4_Ge_6_ (Ln = Y, Ce, Pr, Nd, Sm, Gd–Dy) were reinvestigated,^20,21^ and isostructural Yb_3_Pt_4_Si_6–*x*_^22^ was also discovered. What is interesting about Ln_3_Pt_4_Ge_6_ is that the atoms on the Ln site construct frameworks of both a tilted square lattice and a triangular lattice. Measurements of the magnetic properties of these materials revealed that only Gd_3_Pt_4_Ge_6_ and Tb_3_Pt_4_Ge_6_ were antiferromagnetically ordered while others were paramagnetic.^20^ A pure phase of polycrystalline Dy_3_Pt_4_Ge_6_ could not be synthesized, and thus its magnetic properties were not reported.^20^

Here, we replaced Ge with Sb and obtained millimeter-sized single crystals of Dy_3.00(1)_Pt_2_Sb_4.48(2)_ with crystallographic disorder on both Dy and Sb sites. Dy_3.00(1)_Pt_2_Sb_4.48(2)_ appears to be a new material which crystallizes in a structural type similar to that of RE_3_Pt_4_Ge_6_. By performing magnetic property and electrical transport measurements, we observed complex magnetic behavior in Dy_3.00(1)_Pt_2_Sb_4.48(2)_, including strong magnetic anisotropy, competing AFM sublattices, and multiple metamagnetic transitions. We believe that Dy_3.00(1)_Pt_2_Sb_4.48(2)_ will provide a platform for investigating the relationship between crystallographic disorder and magnetism as well as frustrated magnetism.

## Experimental Details

### Single-Crystal Growth

**Figure 1:**
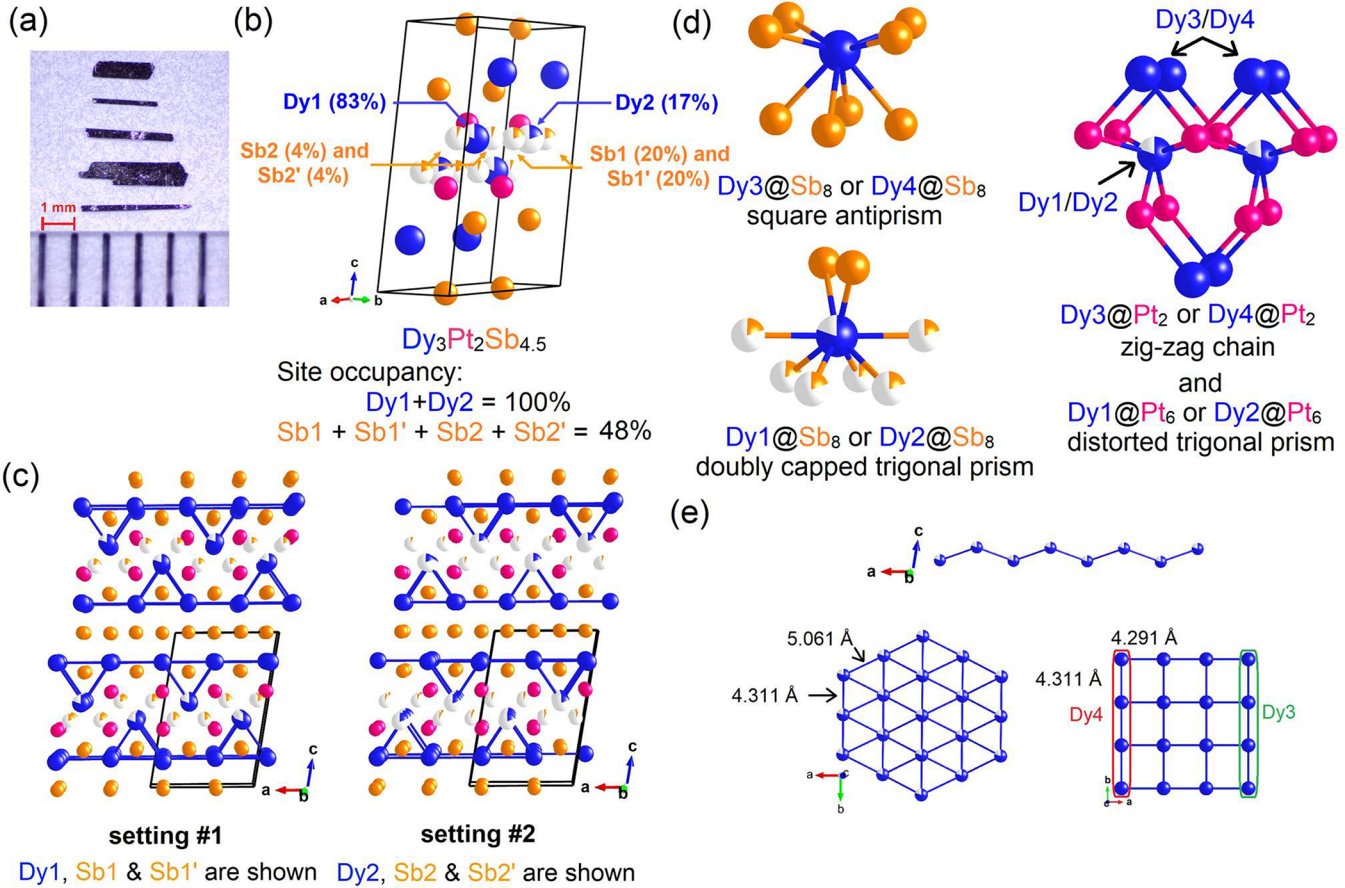
(a) Pictures of the Dy_3.00(1)_Pt_2_Sb_4.48(2)_ crystals under an optical microscope. The composition is from single-crystal X-ray diffraction results. (b) Unit cell and structure of Dy_3.00(1)_Pt_2_Sb_4.48(2)_ with all disordered sites where blue, red, and orange spheres represent Dy, Pt, and Sb atoms, respectively. (c) The two different settings of Dy_3.00(1)_Pt_2_Sb_4.48(2)_ with distinct disordered sites of Dy and Sb. (d) The coordination environment of Dy represented by setting #1. (e) Dy–Dy frameworks represented by setting #1.

The self-flux method with excess Sb was employed to obtain single crystals of Dy_3.00(1)_Pt_2_Sb_4.48(2)_. Dy powder (99.9%, ∼400 mesh, Alfa Aesar), Pt powder (>99.9%, ∼325 mesh, Thermo Scientific), and Sb powder (99.5%, ∼100 mesh, Thermo Scientific) were mixed in a molar ratio of 3:2:50 in an alumina crucible in an Ar-filled glovebox. This crucible was put into a quartz tube, with quartz wool and some glass pieces above to assist the separation of the Sb flux from the crystals after cooling. The tube was purged with argon and then evacuated (<5 × 10^–3^ Torr) and sealed. It was heated in a box furnace to 900 °C and held for 12 h, followed directly by another heat treatment at 1100 °C for 2 days. After 1 day of heating at 1100 °C, the tube was gently agitated, during which the temperature within did not drop below 1000 °C. The furnace was slowly cooled at a rate of 1 °C per hour to 750 °C, where it remained for a few hours, and then the Sb flux was centrifuged out. Water quenching the tube afterward solidified the Sb flux in the wool plug for simple separation from the crystals. Striped crystals with dimensions of up to 0.1 × 0.25 × 3 mm^3^ were obtained, as shown in Figure 1(a). The crystals are stable in air.

### Structure and Phase Determination

The single-crystal X-ray diffraction studies were carried out on a Bruker Kappa Photon III CPAD diffractometer equipped with Mo Kα radiation (λ = 0.71073 Å). A 0.125 mm × 0.093 mm × 0.043 mm piece of a metallic silver block was mounted on a MiTeGen micromount with Paratone 24EX oil. Data were collected in a nitrogen gas stream at 125(2) K using ϕ and ϖ scans. The crystal-to-detector distance was 50 mm using a variable exposure time (2–5 s) depending on θ with a scan width of 1.0°. Bruker SMART software with corrections for Lorentz and polarization effects included was utilized for data requisition. Numerical absorption correction was applied based on crystal-face indexing using *XPREP*. The direct method and full-matrix least squares on F^2^ procedure within the SHELXTL package were employed to solve the crystal structure.^23,24^ Powder X-ray diffraction patterns on crushed crystals, obtained with a Bruker D2 Phaser diffractometer with Cu Kα radiation and a LYNXEYE-XE detector, were consistent with the structure determined by single-crystal diffraction.

### Scanning Electron Microscopy (SEM) Energy-Dispersive X-ray Spectroscopy (EDS)

High-vacuum SEM (ThermoFisher Apreo HiVac with EDAX Elite 150 SDD EDS detector) was used to determine the chemical composition. Samples were mounted on carbon tape before being loaded into the SEM chamber. Multiple points and areas were examined from each sample to get the Dy:Pt:Sb ratio. Samples were analyzed at 20 kV, and the spectra were collected for 100 s to get the chemical composition via APEX EDS software.

### Physical Property Measurement

Oriented single-crystal samples were utilized for physical property measurements. The magnetic properties were measured on a Quantum Design SQUID magnetometer from 2 to 300 K under an external magnetic field of 0–7 T. The electrical transport measurement was conducted on a Quantum Design PPMS from 2.5 to 300 K under an external magnetic field of 0–9 T using a four-probe method.

### Electronic Structure Calculations

Calculations of the crystal orbital Hamiltonian population (COHP) were performed by the tight-binding, linear muffin-tin orbital atomic spheres approximation (TB-LMTO-ASA) using the Stuttgart code.^25−27^ A mesh of 1000 *k* points was used to generate all integrated values.^28^ The number of irreducible *k* points is 70. An energy difference of 0.05 meV was set as the convergence criterion. In the ASA method, the space was filled with overlapping Wigner–Seitz (WS) spheres. The symmetry of the potential was treated as spherical in each WS sphere, with a combined correction on the overlapping part. The WS radii were 3.67 Å for Dy, 2.59 Å for Pt, and 3.30 Å for Sb. Empty spheres were required for the calculation, and the overlap of WS spheres was limited to no more than 16%.

The band structure and density of states (DOS) were calculated by using WIEN2k, which employs the full-potential linearized augmented plane wave method (FP-LAPW) with local orbitals implemented.^29,30^ The electron exchange-correlation potential used was the generalized gradient approximation.^31^ The conjugate gradient algorithm was applied. Reciprocal space integrations were completed over a 5 × 10 × 3 Monkhorst–Pack *k*-point mesh.^32^ Orbital potentials (U = 4 eV) were employed for the Dy *f* electrons. With these settings, the calculated total energy converged to less than 0.1 meV per atom.

## Results and Discussion

### Crystal Structure Determination

By performing single-crystal X-ray diffraction, the formula of the striped crystals was determined to be Dy_3.00(1)_Pt_2_Sb_4.48(2)_, which crystallizes in the monoclinic space group *P*2_1_/*m* (no. 11, *mP*19) and is found to be a new material in the Dy–Pt–Sb ternary system. The crystallographic data, including atomic positions, site occupancies, and refined anisotropic displacement parameters (and equivalent isotropic thermal displacement parameters), are listed in Tables 1, 2, and 3. In our crystallographic model, the best solution emerged when multiple disordered sites were introduced, as shown in Figure 1(b) where 12 disordered sites can be found and 6 of them (Dy1, Sb1, Sb1′ and Dy2, Sb2, Sb2′) are symmetrically equivalent, with the rest correlated by the 2_1_ symmetry. Here we correlate Dy1 with Sb1 and Sb1′ as well as Dy2 with Sb2 and Sb2′, indicating that the Dy1(2) site exists only when Sb1(1) and Sb1′(2′) appear. The interatomic distances between Dy1/Dy2 and Sb2(Sb2′)/Sb1(Sb1′) are ∼1.29 Å. These are not possible bond lengths as they are far below 50% of the common Dy–Sb bond lengths, for example, 3.0660 (2) Å in DySb.^33,34^ Moreover, upon removal of either set of Dy and Sb atoms, a large residual peak and hole can appear, as can be seen in Table S1. Therefore, in order to obtain reasonable crystallographic refinement results, two constraints must be applied to the crystallographic model: 1. The sum of the occupancies of Dy1 and Dy2 was set to be 100%. 2. The occupancies of sites Sb1, Sb1′, Sb2, and Sb2′ were set to be correlated and relaxed. Under such a condition, Sb1 and Sb1′ possessed higher occupancies (∼20% for each site) than Sb2 and Sb2′ (∼4% for each site) and the interatomic distances between Dy1–Sb1 (3.023 (3) Å) and Dy2–Sb2 (3.02 (2) Å) pairs are comparable to the literature on compounds in the Dy–Sb binary system and elemental Sb.^33,34^ The bond lengths of Sb1–Sb1′ (2.579 (4) Å) and Sb2–Sb2′ (2.60 (3) Å) are smaller than in the literature.^35^ Considering that such Sb pairs exist as split interstitials, which can usually lead to short bond length, the short Sb–Sb bond length in this work is reasonable. Under such constraints, the crystallographic disorder with very short Dy–Sb distances can thus be interpreted in a reasonable way such that the Dy1, Sb1, and Sb1′ sites are occupied alternately with the Dy2, Sb2, and Sb2′ sites based on the site occupancies shown in Table 2. A comparison of crystallographic refinement results with different models is also summarized in Table S1. The inclusion of Sb2(Sb2′) and Dy2 significantly improves the results. As shown in Figure 1(c), sites Dy1 and the Sb1 and Sb1′ pair make up setting #1 and Dy2 and the Sb2 and Sb2′ pair make up setting #2. The same coordination environment of Dy can be found in both settings. Two types of Dy@Sb_8_ polyhedra are shown on the left of Figure 1(d). Additionally, when coordinated with Pt atoms, the partially occupied Dy forms Dy@Pt_6_ trigonal prisms while fully occupied Dy constructs the Dy@Pt_2_ zigzag chain. When bonded together, the fully occupied Dy atoms exhibit a distorted square net framework, as shown in Figure 1(e). Moreover, partially occupied Dy1 atoms construct a puckered triangular lattice. The Dy framework can be seen as extending 2D within the *ab* plane and stacking along the *c* axis.

**Table 1:** Single Crystal Structural Refinement for Dy_3.00(1)_Pt_2_Sb_4.48(2)_ at 273(2) K

Refined Formula	Dy_3.00(1)_Pt_2_Sb_4.48(2)_
F.W. (g/mol)	1423.14
space group; *Z*	*P*2_1_/*m*; 2
*a* (Å)	8.6252(8)
*b* (Å)	4.3109(3)
*c* (Å)	12.968(1)
β (deg)	99.609(3)
*V* (Å^3^)	475.43(7)
extinction coefficient	0.00037(5)
θ range (deg)	3.090–31.902
no. of reflections; *r*_*int*_	23877; 0.0329
no. of independent reflections	1699
no. of parameters	75
*R*_1_*: ωR*_2_ (*I* > 2δ(*I*))	0.0272; 0.0432
goodness of fit	1.151
diffraction peak and hole (e^–^/Å^3^)	2.954; −2.613

**Table 2:** Atomic Coordinates and Equivalent Isotropic Displacement Parameters for Dy_3.00(1)_Pt_2_Sb_4.48(2)_ at 273(2) K^a^

Atom	Wyck.	Occ.	*x*	*y*	*z*	U_*e*__*q*_
Pt1	2*e*	1	0.52636 (4)	1/4	0.61894 (3)	0.0044 (1)
Pt2	2*e*	1	0.03314 (4)	1/4	0.61904 (3)	0.0045 (1)
Dy1	2*e*	0.830 (1)	0.23486 (7)	1/4	0.43955 (5)	0.0042 (1)
Sb1	2*e*	0.200 (3)	0.4155 (4)	3/4	0.5623 (3)	0.0051 (7)
Sb1′	2*e*	0.201 (3)	0.1156 (4)	3/4	0.5621 (3)	0.0051 (7)
Dy2	2*e*	0.170 (1)	0.2648 (3)	3/4	0.5606 (2)	0.0042 (1)
Sb2	2*e*	0.039 (2)	0.384 (2)	1/4	0.439 (1)	0.0051 (7)
Sb2′	2*e*	0.039 (2)	0.083 (2)	1/4	0.437 (1)	0.0051 (7)
Dy3	2*e*	1	0.57655 (6)	3/4	0.81070 (4)	0.0050 (1)
Dy4	2*e*	1	0.07890 (6)	3/4	0.81115 (4)	0.0050 (1)
Sb3	2*e*	1	0.12475 (7)	1/4	0.99840 (5)	0.0053 (1)
Sb4	2*e*	1	0.37569 (7)	3/4	0.00186 (5)	0.0054 (1)
Sb5	2*e*	1	0.31256 (7)	1/4	0.74994 (5)	0.0051 (1)
Sb6	2*e*	1	0.81150 (7)	1/4	0.74621 (5)	0.0049 (1)

a U_*eq*_ is defined as one-third of the trace of the orthogonalized U_*ij*_ tensor (Å^2^).

**Table 3:** Anisotropic Thermal Displacement Parameters for Dy_3.00(1)_Pt_2_Sb_4.48(2)_

Atom	U_11_	U_22_	U_33_	U_23_^a^	U_13_^a^	U_12_^a^
Pt1	0.0050 (2)	0.0040 (2)	0.0049 (2)	0	0.0009 (1)	0
Pt2	0.0049 (2)	0.0042 (2)	0.0049 (2)	0	0.0009 (1)	0
Dy1	0.0020 (2)	0.0030 (2)	0.0048 (2)	0	0.0013 (2)	0
Sb1	0.003 (2)	0.004 (2)	0.008 (2)	0	0.0003 (10)	0
Sb1′	0.005 (2)	0.005 (2)	0.008 (2)	0	0.002 (1)	0
Dy2	0.019 (1)	0.011 (1)	0.009 (1)	0	–0.0003 (9)	0
Sb2	0.021 (9)	0.019 (9)	0.016 (8)	0	0.006 (6)	0
Sb2′	0.016 (8)	0.012 (8)	0.008 (8)	0	0.001 (5)	0
Dy3	0.0057 (2)	0.0045 (2)	0.0053 (2)	0	0.0011 (1)	0
Dy4	0.0056 (2)	0.0043 (2)	0.0054 (2)	0	0.0010 (1)	0
Sb3	0.0061 (3)	0.0050 (3)	0.0056 (3)	0	0.0012 (2)	0
Sb4	0.0057 (3)	0.0053 (3)	0.0057 (3)	0	0.0012 (2)	0
Sb5	0.0043 (3)	0.0048 (3)	0.0067 (3)	0	0.0013 (2)	0
Sb6	0.0043(3)	0.0045 (3)	0.0064 (3)	0	0.0013 (2)	0

a For an explanation of the anisotropic thermal displacement parameters, see ref (39).

To confirm the chemical composition, SEM-EDS measurements were performed on two different crystals from different batches. The EDS results averaged from five points are shown in Table S2 in the Supporting Information. The average formula was determined to be Dy_3.1(3)_Pt_2.0(7)_Sb_3.8(4)_ when normalized to the concentration of Pt, which is consistent with the results from the single-crystal X-ray diffraction measurement. Moreover, the powder X-ray diffraction pattern of the crystals is illustrated in Figure S1, which implies high purity of the crystals. A preferred orientation of (00l) is clearly seen in the powder XRD pattern.

### Structural Relations and Bonding Analysis

**Figure 2:**
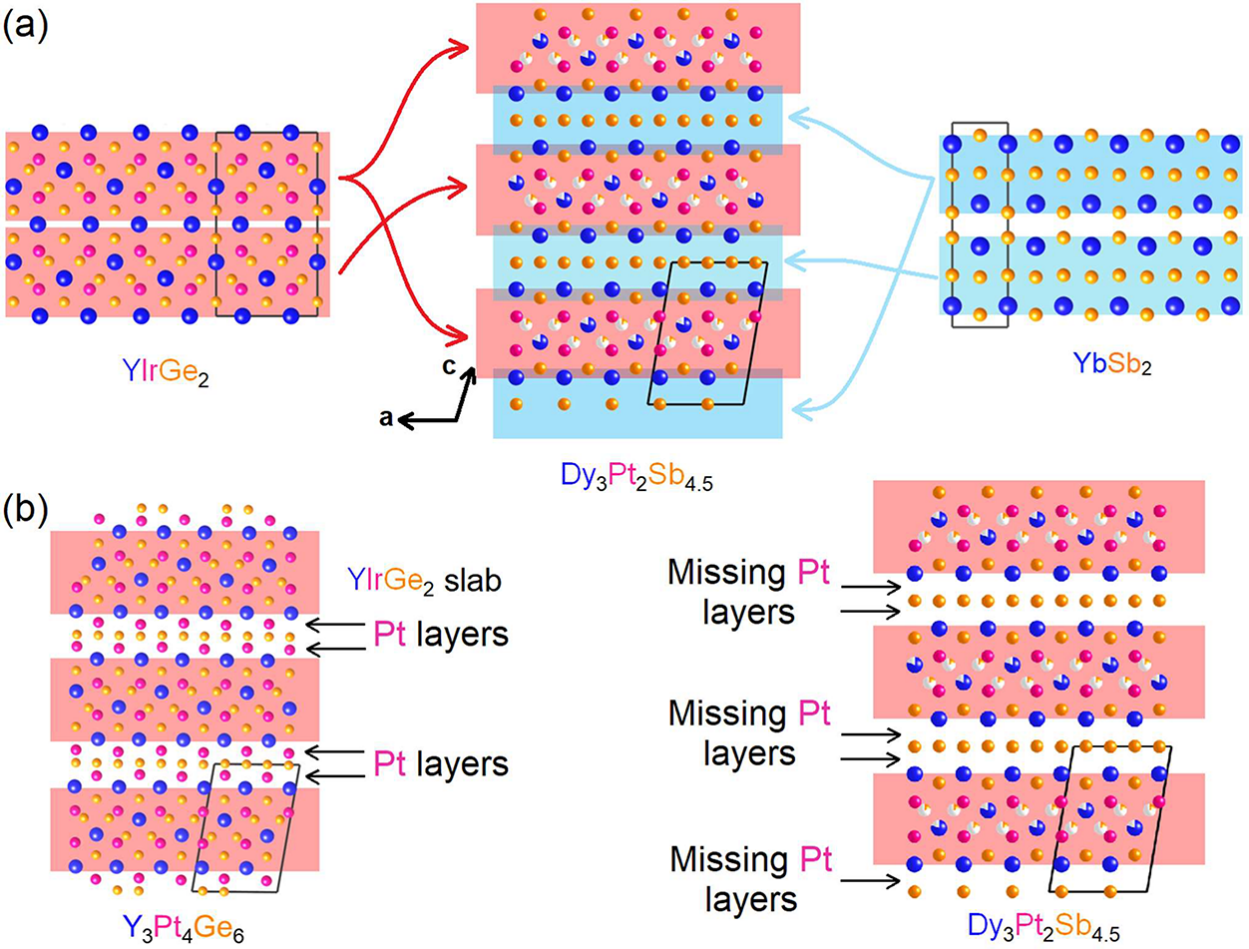
(a) Relation among structural motifs of YIrGe_2_ (left), Dy_3_Pt_2_Sb_4.48_ (middle), and YbSb_2_ (right). The shaded parts of the same color are similar to each other, and the arrows indicate the location of the intergrowth. (b) Comparison between the structures of Y_3_Pt_4_Ge_6_ and Dy_3_Pt_2_Sb_4.48_. The red shades stand for the common YIrGe_2_ slabs in both structural types. The arrows indicate where the absences of Pt atoms occur.

When several repeats of the unit cell along the *c* axis are considered, certain structural motifs within previously reported structures can be identified. Both YIrGe_2_ and YbSb_2_ have a top and bottom motif that are symmetrical about a mirror plane through the center of the unit cell horizontally, Figure 2(a) (left) and (right), respectively. Setting #1 of Dy_3.00(1)_Pt_2_Sb_4.48(2)_ can be seen as the intergrowth of alternating slabs of YIrGe_2_ and YbSb_2_ along the *c* axis, as shown in Figure 2(a) (middle). The adjacent YIrGe_2_ and YbSb_2_ slabs share a layer of Dy and Sb in Dy_3.00(1)_Pt_2_Sb_4.48(2)_. As for setting #2 of Dy_3.00(1)_Pt_2_Sb_4.48(2)_, the order of the top and bottom halves of YIrGe_2_ switches, which is not shown here. This repeating motif in Dy_3.00(1)_Pt_2_Sb_4.48(2)_ is similar to repeat slabs in Y_3_Pt_4_Ge_6_ for which previous reports^19^ claimed to be built from alternating YIrGe_2_-type and ThCr_2_Si_2_-type^36,37^ slabs. However, as can be seen in Figure 2(b), the alternating layer in Y_3_Pt_4_Ge_6_ contains two layers of Pt atoms which are missing in Dy_3.00(1)_Pt_2_Sb_4.48(2)_.

**Figure 3:**
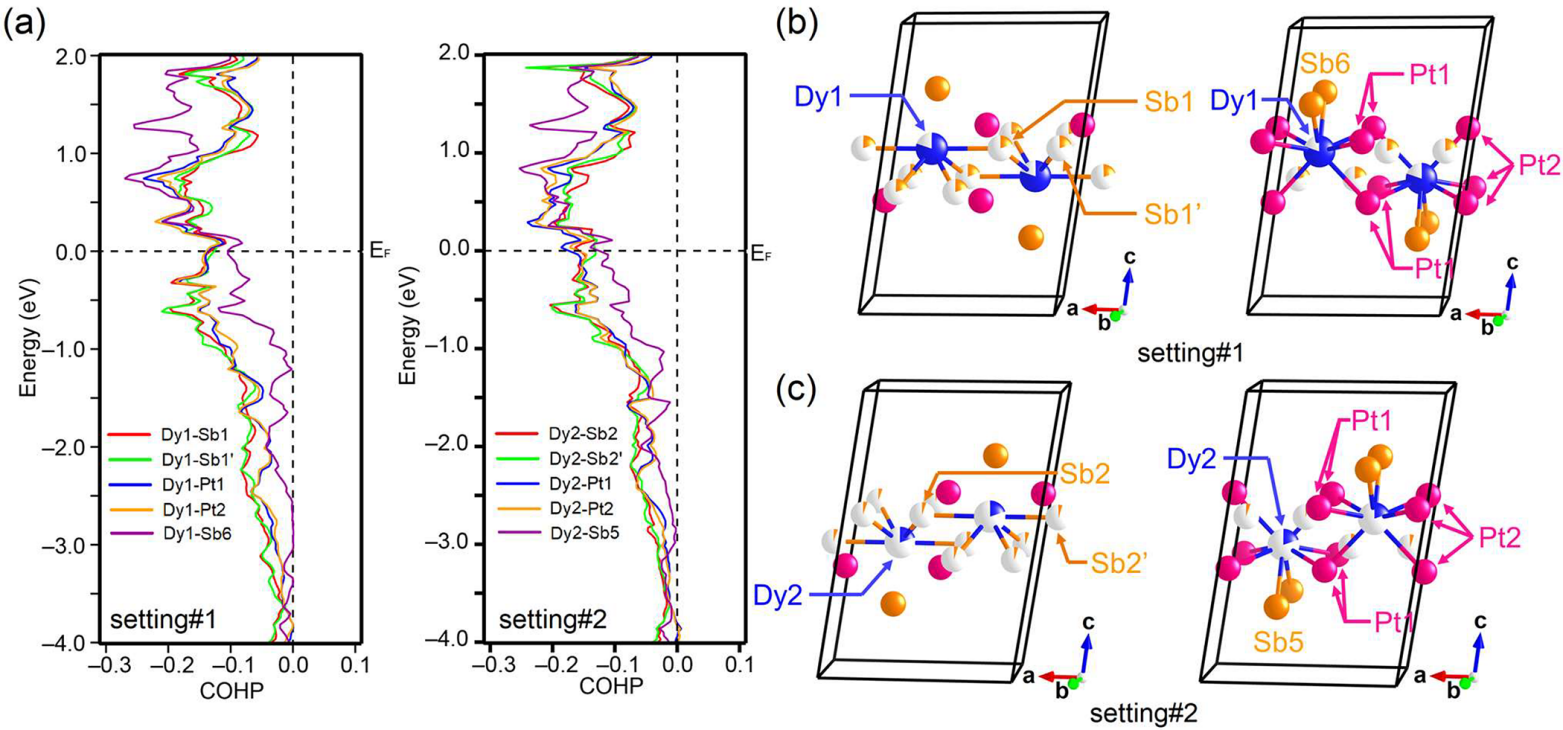
(a) COHP curves for both settings of Dy_3_Pt_2_Sb_4.48_. (b) and (c) Visualization of related chemical bonds mentioned in (a).

To better understand how crystallographic disorder affects the structure of Dy_3.00(1)_Pt_2_Sb_4.48(2)_, COHP calculations were performed for both settings. The relevant chemical bonds for disordered Dy1 and Dy2 are visualized in Figure 3(b) and (c), respectively. All bond lengths were set to be no longer than 3.5 Å. Figure 3(a) presents the COHP curves for both settings where the positive part of the *x* axis indicates a bonding interaction and the negative part shows an antibonding interaction. Similar behaviors can be found for Dy1(Dy2)-Sb1(Sb2) and Dy1(Dy2)-Sb1′(Sb2′) as well as Dy1(Dy2)-Pt1 and Dy1(Dy2)-Pt2. The disordered Dy in both settings shows nearly all antibonding features when bonded with the surrounding atoms within the visible energy range (−4 to 2 eV). Such behavior indicates that the disordered Dy1 and Dy2 sites both destabilize the structure from −4 to 2 eV, which might explain why they are both partially occupied. More COHP curves for Dy–Pt, Dy–Sb, Pt–Sb, and Sb–Sb bonds can be found in Figure S2 in the Supporting Information, which demonstrates significant antibonding interaction above ∼−2.5 eV.

### Magnetic Characterization

**Figure 4:**
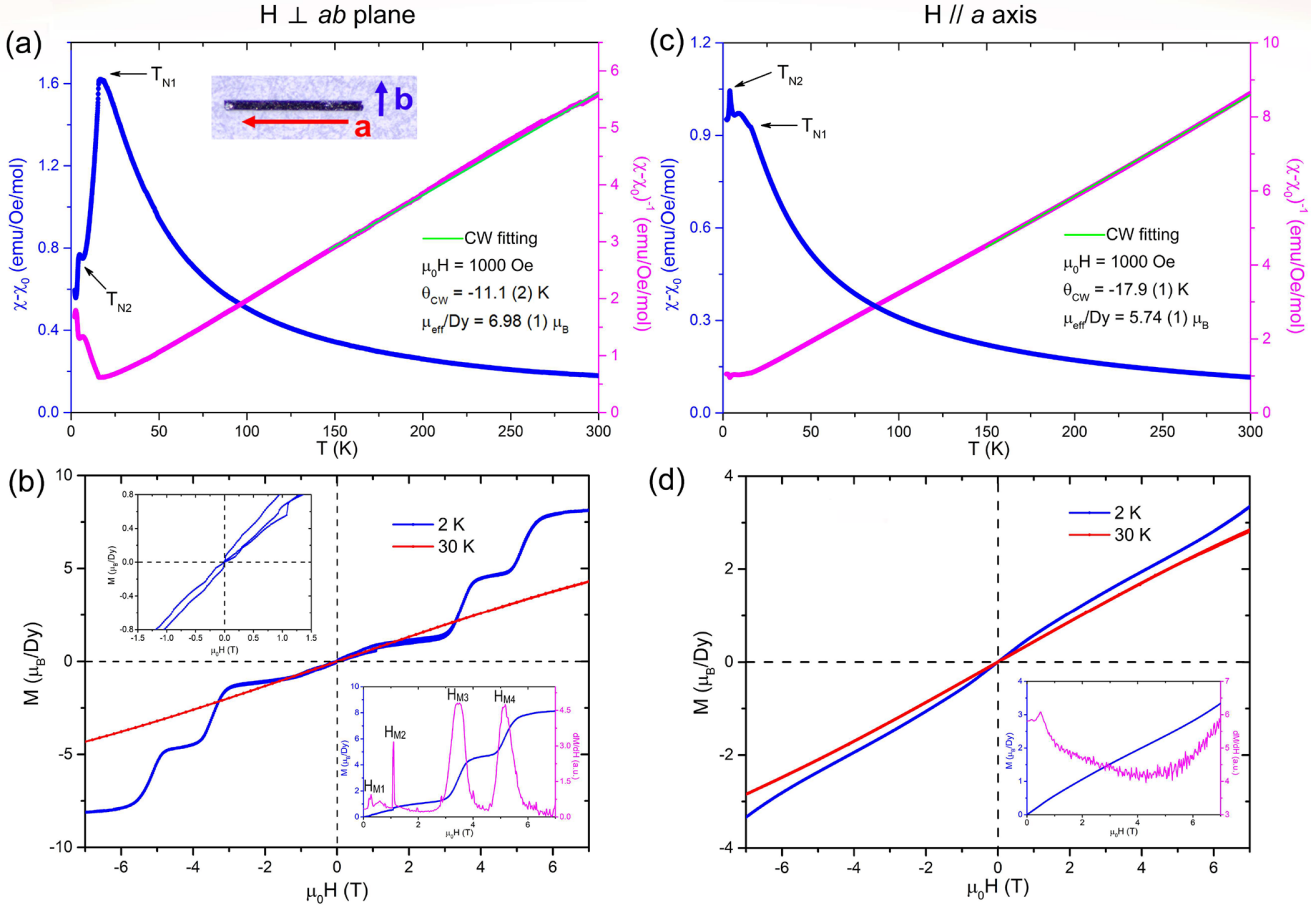
Temperature dependence of magnetic susceptibility and inverse magnetic susceptibility when the external magnetic field is (a) perpendicular to the *ab* plane or (c) parallel to the *a* axis. The inset of (a) is a picture of a crystal shown the direction of crystallographic axes. (Main panel) Hysteresis loop of Dy_3_Pt_2_Sb_4.48_ under various temperatures when the external magnetic field is (b) perpendicular to the *ab* plane or (d) parallel to the *a* axis. The inset figures in (b) are (top left) an enlarged hysteresis loop at 2 K from −1.5 to 1.5 T and (bottom right) an M vs H curve from 0 to 7 T with its first derivative. The inset in (d) is the M vs H curve from 0 to 7 T with its first derivative.

Magnetic property measurements were performed on Dy_3.00(1)_Pt_2_Sb_4.48(2)_ single crystals with the magnetic field applied along two directions: perpendicular to the *ab* plane (H⊥*ab*) and parallel to the *a* axis (H//*a*). The orientation of a representative stripelike crystal is shown in the inset of Figure 4(a). The temperature dependence of magnetic susceptibility (χ) and inverse χ under an applied magnetic field of 1000 Oe from 2 to 300 K are shown in Figure 4(a) and (c). Both curves were collected under field-cooling (FC) history. We observed distinct magnetic anisotropy from the data presented in Figure 4(a) and (b). When the field (H) is perpendicular to the *ab* plane (Figure 4(a)), an AFM transition can be found around T_N1_ ≈ 17 K while another peak is seen at T_N2_ ≈ 4.5 K. The magenta curve in Figure 4(a) shows the temperature dependence of 1/χ. When the external magnetic field is parallel to the *a* axis, the χ versus T curve exhibits different behavior. As shown in the main panel of Figure 4(c), the magnetic susceptibility increases with decreasing temperature until a kink is seen at ∼15.9 K, indicating that the spin easy axis is within the *ab* plane. Upon further cooling, another sharp peak appears at ∼3.9 K. We fitted the 1/χ data using the modified Curie–Weiss (CW) law,
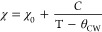
where χ is the magnetic susceptibility of the material, χ_0_ and *C* are independent of temperature (with the latter related to the effective moment and the former related to the core diamagnetism and temperature-independent paramagnetic contributions such as Pauli paramagnetism), and θ_CW_ is the Curie–Weiss constant. The best fit for both directions was obtained in the temperature range of 150–300 K. θ_CW_ obtained from the fit is ∼−11.1 (2) K when H⊥*ab* and ∼−17.9 (1) K when H//*a*; the negative sign suggests antiferromagnetic interaction within the fitted temperature range. The effective moment (μ_eff_) per Dy is calculated by μ_eff_/Dy = √8*C*/3 μ_B_, which leads to 6.98 (1) μ_B_/Dy when H⊥*ab* and 5.74 (1) μ_B_/Dy when H//*a*, both of which are far below the expected values of Dy^2+^ (10.6 μ_B_), Dy^3+^ (10.65 μ_B_), and Dy^4+^ (9.72 μ_B_).The small μ_eff_ may be due to the intermediate *f*-electron configuration of Dy in our material.^38^ The difference between μ_eff_ from the two directions may originate from the strong magnetic anisotropy.

To further understand the magnetic behaviors of Dy_3.00(1)_Pt_2_Sb_4.48(2)_, the hysteresis loops were measured as shown in Figure 4(b) and (d). For H⊥*ab*, several metamagnetic transitions can be seen in Figure 4(b). To better visualize it, a plot from 0 to 7 T for the first cycle of the loop and its first derivative is placed in the bottom-right corner of Figure 4(b). Four obvious peaks at H_M1_ ≈ 0.25 T, H_M2_ ≈ 1.1 T, H_M3_ ∼ 3.5 T, and H_M4_ ∼ 5.1 T can be found in the derivative curve under 2 K, which correspond to four metamagnetic transitions. The other inset in the top-left corner is the enlarged region between −1.5 and 1.5 T for the loop. No remanent magnetization can be found, and it indicates that there is no significant ferromagnetic component in Dy_3.00(1)_Pt_2_Sb_4.48(2)_, i.e., spins are not canted. Under 2 K, the magnetization at 7 T is ∼7.7 μ_B_/Dy. When the temperature is raised to 30 K, a nearly linear relation can be observed. Similarly, the measurement of the hysteresis loop in the other field direction (H//*a*) was also performed. Not surprisingly, strong magnetic anisotropy leads to completely different features for H//*a*. No obvious metamagnetic transition can be seen in Figure 4(d). Instead, two slope changes for the hysteresis loop measured under 2 K are observed, evidenced by the magenta dM/dH curve in the inset. The hysteresis loop does not show any trend to saturate when H//*a* is at 2 K.

### Electrical Transport Measurements

**Figure 5:**
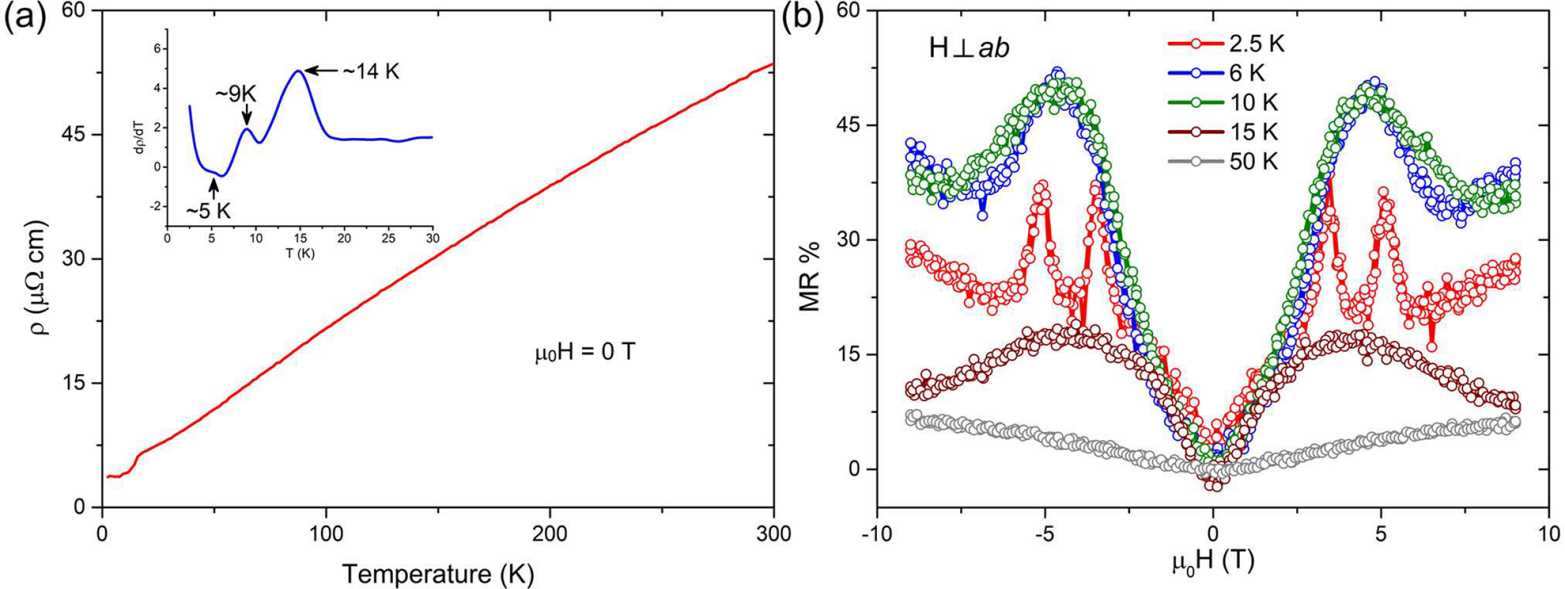
(a) (Main panel) Temperature-dependent electrical resistivity with no magnetic field applied. (Inset) First derivative of the main panel figure. (b) Magnetoresistance measured from −9 to 9 T at various temperatures when the magnetic field is applied perpendicularly to the *ab* plane.

Figure 5(a) and (b) illustrates the temperature-dependent resistivity and magnetoresistance of Dy_3.00(1)_Pt_2_Sb_4.48(2)_. When no magnetic field is applied, the electrical resistivity of the material decreases with decreasing temperature, indicating a metallic nature, which is further supported by the electronic structure shown in Figure S2. A kink is found at ∼15.5 K in the main panel of Figure 5(a), which reflects the magnetic transition observed at T_N1_ in Figure 4(a) and (c). When plotting the first derivative of the ρ vs T curve, two features at ∼5 K and ∼9 K can be found. The 5 K transition corresponds to the magnetic transition at T_N2_ while the 9 K transition might originate from another magnetic transition that was not clearly observed in our magnetic measurement. When the magnetic field is applied perpendicularly to the *ab* plane, two peaks, one at ∼3.5 and one at ∼5.1 T, can be observed for the magnetoresistance curve at 2.5 K in Figure 5(b). The two peaks are consistent with the two metamagnetic transitions seen in Figure 4(b) at H_M3_ and H_M4_. Although more features are observed under a lower field in the magnetic hysteresis loop for H⊥*ab*, no obvious transitions can be found in the magnetoresistance measurements. This is primarily due to the intrinsically low resistivity of the sample, and thus the low-field metamagnetic transitions are possibly obscured by the noise. Interestingly, when the system is warmed up to 6 K, which is above the T_N1_, the peaks at H_M3_ and H_M4_ disappear while a broad peak emerges at ∼4.5 T which persists until 15 K and disappears at 50 K. The broad peak may originate from another metamagnetic transition corresponding with T_N1_.

Based on the findings above, a brief discussion of the magnetic properties of Dy_3.00(1)_Pt_2_Sb_4.48(2)_ is made here. Two magnetic transitions are found in the temperature-dependent magnetic susceptibility curves for both directions at T_N1_ ≈ 3.8 K and T_N2_ ≈ 15 K. However, the higher-temperature one is more predominant when H⊥*ab*, but when H//*a*, the lower-temperature one prevails. Considering the distinct fitted θ_CW_, we speculate that there are two AFM sublattices in Dy_3.00(1)_Pt_2_Sb_4.48(2)_ which correspond to T_N1_ and T_N2_ in χ vs T curves, i.e., AFM1 that corresponds to T_N1_ and AFM2 that corresponds to T_N2_. Complex interactions between the two AFM sublattices may lead to multiple metamagnetic transitions in the hysteresis loops at 2 K. Supported by the transport measurement, the two major metamagnetic transitions at H_M3_ and H_M4_ are correlated with the AFM2 sublattice. Besides the speculation above, another possibility of the magnetic structure could be that one Dy sublattice is responsible for different interaction and coupling constants along different crystallographic directions. In the meantime, the other Dy sublattice results in different magnetic orderings with decreasing temperature, such as spin-reorientations, etc. Based on the complex observation, further studies including X-ray magnetic circular dichroism (XMCD) measurements need to be conducted to better interpret the magnetic structure of Dy_3.00(1)_Pt_2_Sb_4.48(2)_, especially the relationship between the magnetic structure and the two settings of disordered Dy and Sb.

## Conclusions

In this article, motivated by the previously reported material type Y_3_Pt_4_Ge_6_, with both square net and triangular lattices of rare-earth elements, we synthesized millimeter-sized single crystals of a new material Dy_3.00(1)_Pt_2_Sb_4.48(2)_, which crystallizes in a Y_3_Pt_4_Ge_6_-type related structure. Based on measurements of the magnetic properties, we observed two transitions for AFM ordering at ∼15 K and ∼4 K, which might originate from two competing AFM sublattices. Furthermore, strong magnetic anisotropy and metamagnetic transitions were found for Dy_3.00(1)_Pt_2_Sb_4.48(2)_. Therefore, further studies, including XMCD measurements, should be carried out to better understand the complex spin structure of this material. An unusual valence state of Dy was also extrapolated from the Curie–Weiss fitting for the effective moment, which deserves follow-up investigations. The magnetic complexity and crystallographic disorder of Dy_3.00(1)_Pt_2_Sb_4.48(2)_ along with the availability of single crystals provides a platform for investigating magnetism in such structural motifs when Dy is substituted by other rare-earth elements.

## Supporting Information Available

Crystallographic data of the title compound under different constraints; elemental analysis based on EDS; the powder X-ray diffraction pattern; and the proposed magnetic phase diagram (PDF)
