# Supplementary material for: Crystallographic Disorder and Strong Magnetic Anisotropy in Dy3Pt2Sb4.48
Source: Inorg Chem. 2024 Feb 14;63(8):3648–55. doi: 10.1021/acs.inorgchem.3c01850 (PMC10900285; doi:10.1021/acs.inorgchem.3c01850)
Supplement: Supplementary file 1 — ic3c01850_si_001.pdf [file ic3c01850_si_001.pdf]

Supplementary Information

Crystallographic Disorder and Strong Magnetic Anisotropy in $Dy_3Pt_2Sb_{4.48}$

Terry Paske,[a] Yingdong Guan,[b] Chaoguo Wang,[a] Curtis Moore,[c] Zhiqiang Mao,[b] Xin Gui [a]*

[a] Department of Chemistry, University of Pittsburgh, Pittsburgh, PA, 15260, USA
[b] Department of Physics, Pennsylvania State University, University Park, PA, 16801, USA
[c] Department of Chemistry and Biochemistry, The Ohio State University, Columbus, OH, 43210, USA

Table of Contents

Table S1 .... S2

Table S2 .... S3

Figure S1 .... S4

Figure S2 .... S5

Figure S3 .... S6

Table S1. Comparsison of single crystal structure refinement results for $Dy_{3.00(1)}Pt_2Sb_{4.50(2)}$ at 273 (2) K.

Refined Formula	$Dy_{3.00(1)}Pt_2Sb_{4.50(2)}$	No Sb2 and Sb2'	No Sb2/2' and Dy2
F.W. (g/mol)	1424.95	1424.95	1424.95
Space group; Z	$P\ 2_1/m$; 2	$P\ 2_1/m$; 2	$P\ 2_1/m$; 2
a (Å)	8.6252 (8)	8.6252 (8)	8.6252 (8)
b (Å)	4.3109 (3)	4.3109 (3)	4.3109 (3)
c (Å)	12.968 (1)	12.968 (1)	12.968 (1)
β (°)	99.609 (3)	99.609 (3)	99.609 (3)
V ($Å^3$)	475.43 (7)	475.43 (7)	475.43 (7)
Extinction Coefficient	0.00040 (4)	0.00032 (5)	0.00000 (13)
θ range (°)	3.090-31.902	3.090-31.902	3.090-31.902
No. reflections; R_{int}	23877; 0.0329	23877; 0.0329	23877; 0.0329
No. independent reflections	1699	1699	1699
No. parameters	91	79	71
R_1: ωR_2 (I>2δ(I))	0.0257; 0.0392	0.0316; 0.0500	0.0845; 0.1405
Goodness of fit	1.143	1.431	3.971
Diffraction peak and hole (e^-/ $Å^3$)	2.098; -2.257	8.992; -2.445	58.807; -5.725

Table S2. The atomic ratios from Energy-Dispersive X-ray Spectroscopy (EDS) result of two different crystals of $Dy_3Pt_2Sb_{4.5}$.

	**Dy% (Error%)**	**Pt% (Error%)**	**Sb% (Error%)**
**Number of points**		**Sample1**	
**1**	34.71 (3.95)	22.71 (7.93)	42.58 (4.72)
**2**	34.74 (3.88)	22.15 (7.99)	43.11 (4.66)
**3**	34.68 (3.83)	22.02 (7.28)	43.31 (4.65)
**4**	34.89 (3.91)	22.30 (7.54)	42.81 (4.84)
**5**	34.77 (3.92)	22.45 (8.10)	42.78 (4.84)
**Number of points**		**Sample2**	
**1**	34.81 (3.93)	22.99 (7.75)	42.20 (4.90)
**2**	34.86 (3.88)	22.80 (7.38)	42.35 (4.88)
**3**	34.75 (3.85)	22.87 (8.03)	42.38 (4.87)
**4**	35.06 (3.94)	22.63 (7.79)	42.31 (4.86)
**5**	34.99 (3.94)	22.51 (7.86)	42.50 (4.84)
**Average**	34.82 (3.90)	22.54 (7.77)	42.63 (4.81)
**Normalized to Pt**	3.1 (3)	2.0 (7)	3.8 (4)

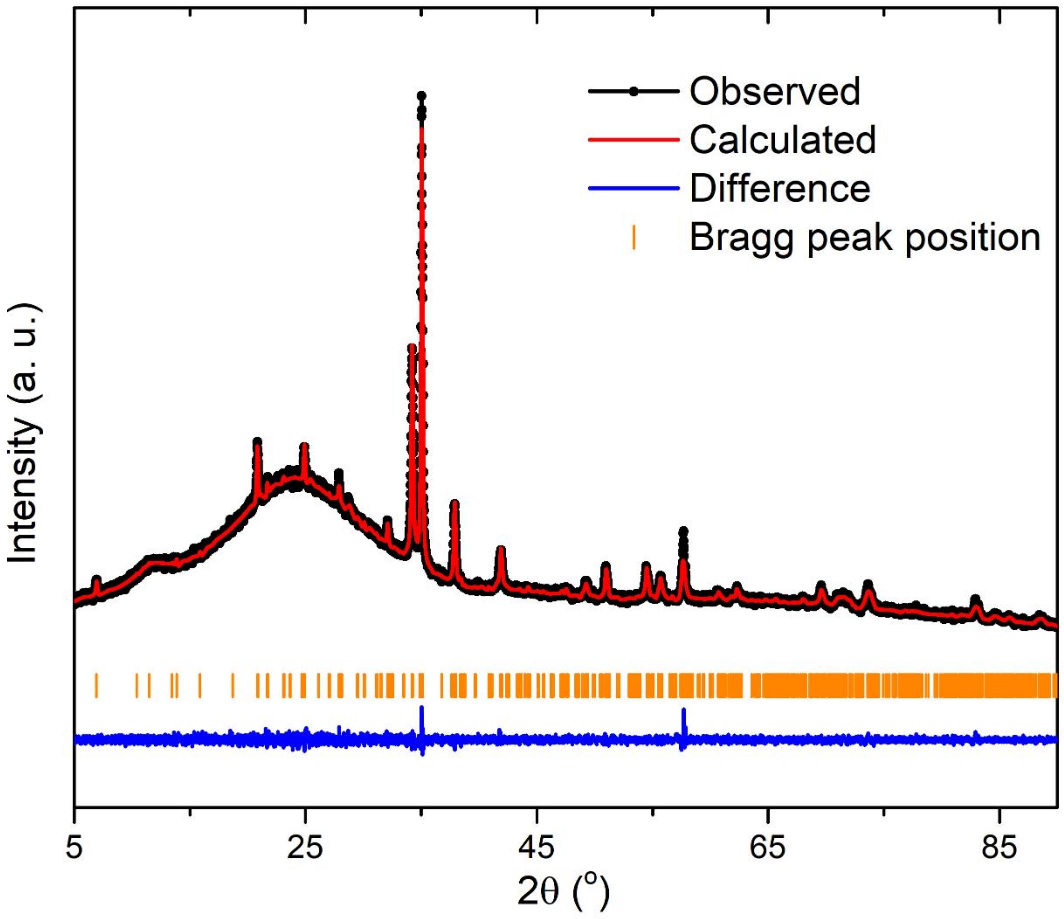

Figure S1. Powder XRD pattern of the crushed crystals of $Dy_{3.00(1)}Pt_2Sb_{4.48(2)}$. Blue and red lines stand for calculated and observed patterns, respectively. The crushed crystals show a preferred orientation of (00l) so that the observed peak for, for instance, (005) peak is much higher than calculated. Therefore, a refinement for preferred orientation of (00l) has been conducted.

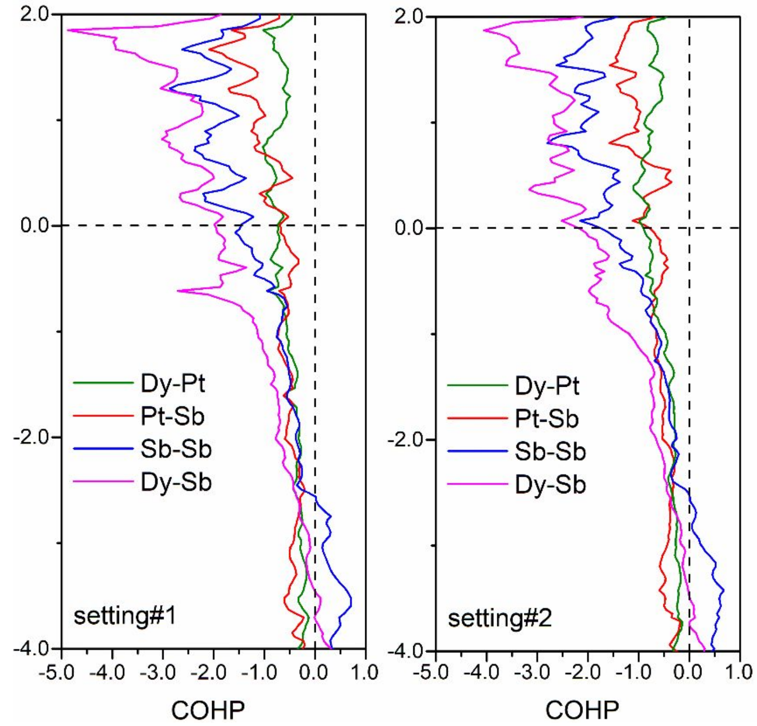

Figure S2. COHP curves of $Dy_{3.00(1)}Pt_2Sb_{4.48(2)}$ for both settings.

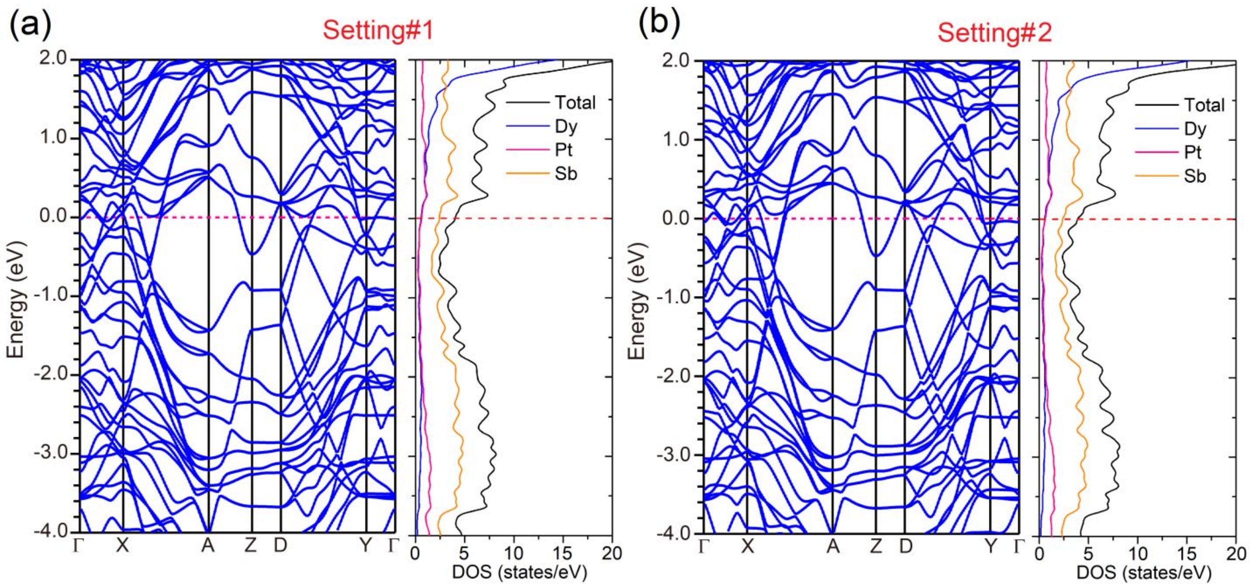

Figure S3. Band structure and DOS of $Dy_{3.1(3)}Pt_{2.0(7)}Sb_{3.8(4)}$ for **(a)** setting#1 and **(b)** setting#2.
